# Harnessing the vasculo-protective potential of microglia

**DOI:** 10.18632/aging.204509

**Published:** 2023-02-01

**Authors:** Sebok K. Halder, Arjun Sapkota, Richard Milner

**Affiliations:** 1 San Diego Biomedical Research Institute, San Diego, CA 92121, USA

**Keywords:** blood-brain barrier integrity, chronic mild hypoxia, microglia, aging, endothelial cells

Blood vessels in the central nervous system (CNS) have unique properties distinct from vessels in other organs, because of their high electrical resistance and low permeability, which creates a highly selective barrier that protects sensitive CNS parenchymal tissue from potentially harmful blood constituents. The molecular basis of this blood-brain barrier (BBB) relies on a combination of structures, including endothelial adherens and tight junction protein complexes, extracellular matrix (ECM) components of the vascular basement membrane, and the influence of adjacent CNS-resident cells such as astrocytes and pericytes [1]. The importance of the BBB is underlined by the fact that most common neurological diseases, including stroke, multiple sclerosis, vascular dementia, and Alzheimer’s disease, have disrupted BBB integrity as the root cause of disease pathogenesis, either in the initiation or maintenance of disease. In addition to these disease conditions, it is now widely accepted that BBB integrity also deteriorates as part of the normal aging process [2, 3].

In the Milner lab we study the influence of hypoxia on cerebral blood vessels for two reasons. First, as hypoxia triggers a strong angiogenic response in cerebral blood vessels that results in greatly increased vessel density as well as upregulation of tight junction proteins, it is important to understand these responses so that we might harness them for therapeutic means. Second, because hypoxia is a key component of many age-related diseases including lung diseases such as asthma, emphysema and chronic obstructive pulmonary disease (COPD), heart failure and cerebrovascular disease, it becomes evident that most people will experience some degree of hypoxia at different times of their life.

While it’s well established that chronic mild hypoxia (CMH, 8% O_2_) promotes a robust angiogenic response, we recently found that it also triggers transient BBB disruption, that is associated with aggregation and activation of microglia around the leaky blood vessels [4]. Importantly, microglial depletion markedly enhanced vascular leak, demonstrating an important vasculo-protective function for microglia in maintaining BBB integrity. As a high integrity BBB is a critical determinant of cerebral health, yet evidence suggests that BBB integrity declines with age [2, 3], we recently examined how aging influences both the extent of hypoxia-induced BBB disruption and the associated vasculo-protective function of microglia. This showed that compared to young (8-10 weeks) mice, the number of hypoxia-induced vascular leaks was greatly amplified (5-10 fold) in aged (20 months) mice in all regions of the brain examined [5]. Interestingly, the areas of brain most susceptible to vascular disruption were the olfactory bulb and the midbrain, which showed numbers of vascular leaks 4-fold higher than other regions. To examine if the vascular leak had any functional consequences, we examined olfactory function using the buried food test. This showed that even under normoxic conditions, aged mice took twice as long as young mice to find buried food and more strikingly, hypoxia dramatically impaired the ability to find hidden food at both ages. The clinical significance of these findings is that loss of smell (anosmia) in humans has been shown to be an early predictor of cognitive decline [6]. Taken together, this raises the possibility that age-related hypoxic insults trigger BBB disruption, which manifests initially as a decline in olfactory function, but would also be a harbinger of further BBB disruption in other brain regions, including those involved in cognition. In the same vein, it was notable that the other brain region most susceptible to hypoxia-induced vascular disruption was the midbrain, particularly the substantia nigra and the red nucleus. This suggests that hypoxia-induced BBB disruption in these areas could negatively impact motor control and coordination, and further imply that hypoxia may be a previously unidentified trigger of movement disorders such as Parkinson’s disease.

When we analysed the impact of aging on microglia activity, we discovered an interesting paradox. On the one hand, microglia in aged brain were far more activated as assessed by morphological criteria and expression of activation markers such as Mac-1 and CD68, but on the other hand, they displayed a marked deficit in the ability to aggregate around leaky blood vessels [5]. These findings are consistent with the work of others who showed microglia in aged brain are typically more activated than in young mice [7]. Interestingly, microglia in the aged brain can be re-programmed by removing all microglia with the colony stimulating factor-1 receptor (CSF-1R) antagonist PLX5622, and then allowing the CNS to repopulate with new microglia displaying a younger phenotype. Notably, this approach was shown to reverse age-related cognitive decline, although vascular integrity was not examined [7]. In our study we took the simpler approach of reducing microglial activation state in the aged brain by treating mice with minocycline and this successfully reduced the number of hypoxia-induced vascular leaks. Based on these data, we proposed a biphasic relationship between microglial activation and vasculo-protection, such that microglia need to become activated to confer protection, but if they become too activated, as in the aged brain, this protection declines [5].

Several outstanding questions remain. First, what are the molecular mechanisms that account for microglial vasculo-protection? To confer benefit to a leaky blood vessel, microglia need to become activated and migrate towards the site of vascular leak and wrap around the leaky blood vessel. We have shown that microglial activation is mediated in part by the interaction between fibrinogen leaking out of the blood vessel and the microglial Mac-1 receptor [8], though for such a critical process, it’s likely that other mechanisms may play a role. Second, once the microglia have migrated and accumulated around the site of leak, how do they mediate vasculo-protection? Do microglia physically enwrap the leaky blood vessel and thus plug the hole, or do they secrete factors that encourage endothelial proliferation or maturation in the form of enhanced endothelial expression of ECM proteins or adherens or tight junction proteins? Third, how can we optimize the vasculo-protective influence of microglia? While re-programming of aged microglia may be a short-term option, it’s not likely to be sustainable in the long term as studies have shown that re-programmed microglia in aged brain are strongly influenced by their environment and quickly revert to their aged phenotype. A more promising approach might be to identify which aspects of microglial behavior promote vasculo-protective activity and which antagonize it. Potential candidates for destructive behavior include members of the matrix metalloproteinase (MMP) family such as MMP-9 and pro-inflammatory cytokines, such as IL-1 and IL-6, while candidates for vasculo-protective factors include ECM proteins such as laminin, cell surface adhesion molecules such as integrins, tissue inhibitors of MMPs (TIMPs), and anti-inflammatory cytokines such as TGF-β1. Lastly, when considering the interplay between blood vessels and microglia, though we have shown that microglial activation state can influence vasculo-protective function and thus vascular integrity, it’s also possible, indeed likely, that vascular integrity can influence microglial activation. This is consistent with our finding that in the aged brain, reduced vascular integrity correlates with enhanced baseline microglial activation state under normoxic conditions [5]. Based on these findings, if we can determine how to improve BBB integrity in the aged, this could lead to a reduced level of microglial activation, and if a leak were to subsequently occur, the now less-activated microglia would be better positioned to facilitate optimal vasculo-protection.
